# IL-22 Binding Protein (IL-22BP) in the Regulation of IL-22 Biology

**DOI:** 10.3389/fimmu.2021.766586

**Published:** 2021-11-16

**Authors:** Lauren A. Zenewicz

**Affiliations:** Department of Microbiology and Immunology, College of Medicine, University of Oklahoma Health Sciences Center, Oklahoma City, OK, United States

**Keywords:** IL-22, IL-22 binding protein, cytokines, inflammation, immune regulation

## Abstract

Cytokines are powerful mediators of inflammation. Consequently, their potency is regulated in many ways to protect the host. Several cytokines, including IL-22, have coordinating binding proteins or soluble receptors that bind to the cytokine, block the interaction with the cellular receptor, and thus prevent cellular signaling. IL-22 is a critical cytokine in the modulation of tissue responses during inflammation and is highly upregulated in many chronic inflammatory disease patients, including those with psoriasis, rheumatoid arthritis, and inflammatory bowel disease (IBD). In healthy individuals, low levels of IL-22 are secreted by immune cells, mainly in the gastrointestinal (GI) tract. However, much of this IL-22 is likely not biologically active due to the high levels of IL-22 binding protein (IL-22BP) produced by intestinal dendritic cells (DCs). IL-22BP is a soluble receptor homolog that binds to IL-22 with greater affinity than the membrane spanning receptor. Much is known regarding the regulation and function of IL-22 in health and disease. However, less is known about IL-22BP. In this review, we will focus on IL-22BP, including its regulation, role in IL-22 biology and inflammation, and promise as a therapeutic. IL-22 can be protective or pathogenic, depending on the context of inflammation. IL-22BP also has divergent roles. Ongoing and forthcoming studies will expand our knowledge of IL-22BP and IL-22 biology, and suggest that IL-22BP holds promise as a way to regulate IL-22 biology in patients with chronic inflammatory disease.

## Introduction

**Figure 1:**
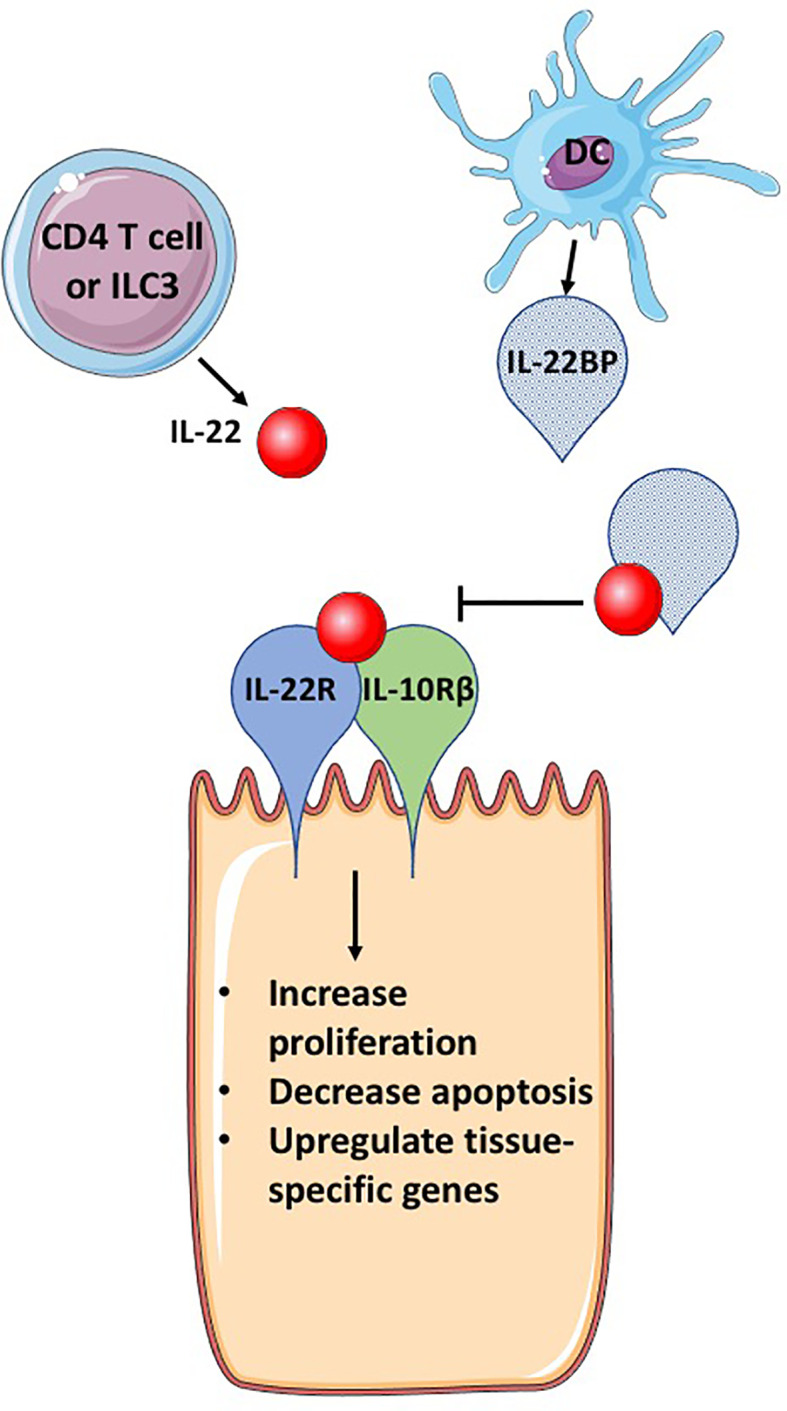
IL-22BP blocks IL-22 signaling. IL-22 is secreted primarily by CD4 T cells and group 3 innate lymphocytes (ILC3s). The receptor for IL-22, a heterodimeric complex of IL-22R and IL-10Rβ, binds IL-22, activating signaling pathways that induce genes important for cellular proliferation, inhibition of apoptosis and tissue specific genes, that can include antimicrobial proteins and mucins. IL-22BP is produced primarily by DCs, is a homolog of IL-22R but lacks a transmembrane domain, allowing for secretion of IL-22BP. IL-22BP binds IL-22 with up to 10,000 greater affinity than IL-22R and therefore in the presence of IL-22BP, most IL-22 binds IL-22BP, inhibiting its ability to bind to the IL-22 receptor complex and cause changes in epithelial cells. This figure is based on our knowledge of IL-22 biology using mouse models. The figure was prepared by modifying Servier Medical Art, licensed under a Common Attribution 3.0 Generic License. http://smart/servier.com/.

Cytokines must be carefully regulated in order to prevent excessive destruction of host tissues. One such mechanism of regulation is through host production of a cytokine-binding protein. Several cytokines have coordinating binding proteins or soluble receptors that bind to the cytokine and prevent cellular signaling by blocking the cytokine’s interaction with its cellular receptor. This property potentially allows for site-specific, microenvironmental regulation of potent cytokines. The best-described examples are IL-18 binding protein and soluble IL-6 and TNF receptors (1–3). IL-22 also has a coordinating binding protein, termed IL-22 binding protein (IL-22BP), that has high homology to one subunit of the heterodimeric IL-22 receptor. IL-22BP binds to IL-22 with greater affinity than the receptor, sequestering IL-22 so it cannot interact with cell surface bound receptor and modulate cell signaling (**Figure 1**). IL-22 is a critical cytokine in tissue responses to inflammation, especially at sites that form a barrier between the environment and the host, such as the skin, lungs, and gastrointestinal (GI) tract (4, 5). IL-22BP has an important role in controlling the biological activity of IL-22 in healthy individuals and during infection or chronic inflammatory diseases. In this review, we will provide a foundation on IL-22 biology and then focus on IL-22BP, including its regulation, role in health and disease, and potential as a therapeutic.

## IL-22 Biology

**Figure 2:**
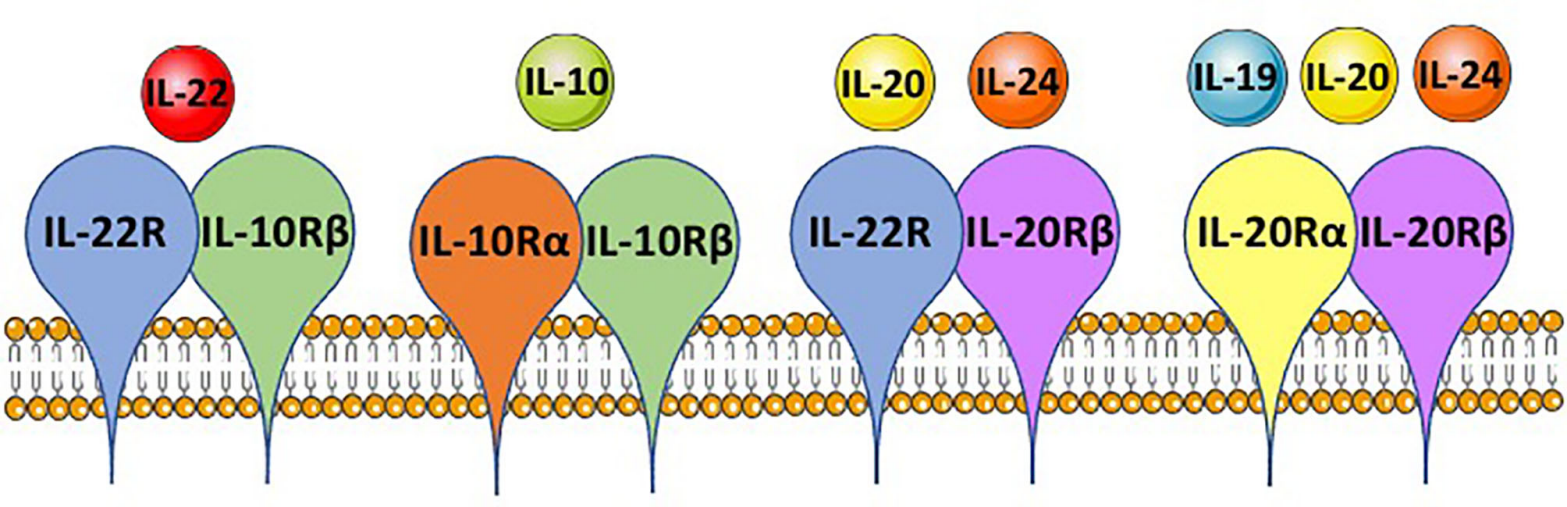
IL-10 family member shared receptor usage. IL-10 family members, IL-10, IL-19, IL-20, IL-22 and IL-24, each bind to a heterodimeric receptor and receptor chains are shared between the cytokines. IL-22 binds to the IL-22R-IL-10Rβ complex, IL-10 binds the IL-10Rα-IL-10Rβ complex, IL-20 and IL-24 both can bind the IL-22R-IL-20Rβ complex, and IL-19, IL-20 and IL-24 can bind the IL-20Rα-IL-20Rβ complex. This is based on mouse immunology. The figure was prepared by modifying Servier Medical Art, licensed under a Common Attribution 3.0 Generic License. http://smart/servier.com/.

IL-22 is a member of the IL-10-related cytokine family, which includes IL-19, IL-20, IL-24, and IL-26 (6). These cytokines were identified from human genome sequencing due to their shared amino acid sequence and structural similarity to IL-10. For signaling, their heterodimeric receptors share several different receptor chains that assort to generate different receptors of different cytokine specificity (6) (**Figure 2**). All cytokine family members are involved to some degree in tissue-mediated responses during inflammation (6). IL-22 is the best-studied member and can contribute to both innate and adaptive immune responses (4, 5). The major sources of IL-22 are CD4 T cells and group 3 innate lymphocytes (ILC3s); the cytokine can also be produced by other immune cells, such as γδ T cells, natural killer (NK) cells, NK T cells, and mucosal associated invariant T (MAIT) cells (4). Like many other cytokines, IL-22 is produced upon immune cell activation, which could be an antigen-specific response to a MHCII-presented peptide for CD4 T cells or innate cytokines, such as IL-23 or IL-1β for ILC3s (5).

IL-22 signals to responsive cells through a heterodimeric receptor composed of IL-22R paired with a second chain, which is IL-10R2 in humans and IL-10Rβ in mice (7, 8). This complex is specific to IL-22 signaling, but each chain can pair with other receptors to form a receptor for related cytokines; IL-10Rα pairs with IL-10Rβ to form the receptor for IL-10, and IL-22R can pair with IL-20Rβ to form one receptor for IL-24 (9) (**Figure 2**). Immune cells normally lack IL-22R and do not respond to IL-22 (10). The main responding cells are epithelial cells, such as keratinocytes, hepatocytes, and enterocytes, but also include other cells, such as fibroblasts (4). In these cells, IL-22 receptor binding leads to phosphorylation of Janus tyrosine kinase (JAK 1) and tyrosine kinase 2 (TYK2), promoting phosphorylation of the transcription factor STAT3. Other pathways have been shown to be activated depending on the cell type and include STAT1, AKT, and MAPKs (4). These signaling pathways lead to activation or repression of many different genes, the best studied of which are highly upregulated by IL-22. Some genes are broadly upregulated in many cell types, such as those that increase cellular proliferation or inhibit apoptosis (11). Many genes upregulated by IL-22 are tissue specific. For example, in the GI tract, IL-22 can induce mucin production in goblet cells or antimicrobial peptides in Paneth cells (12, 13). Many more genes have been shown to be positively and negatively regulated by IL-22 through microarray and RNA sequencing studies, including genes encoding nitric oxide synthase 2 (NOS2), several chemokines (CXCL12, CXCL13, CCL20) and potent systemic secreted factors such as serum amyloid A (SAA) and LPS binding protein (LBP) (4).

IL-22 can have protective or pathogenic roles in inflammation. This dual role has been best elucidated from experimental mouse models with gene-deficient mice or neutralizing antibodies (Abs). This dual-natured activity is thought to be dependent on the context of inflammation, which includes the involved tissue, levels of other cytokines, oxygen and metabolites and their derivatives, and other inflammatory mediators, like leukotrienes and prostaglandins. In general, IL-22 is pathogenic in skin inflammation (14). For example, in psoriasis, IL-22 promotes proliferation of keratinocytes, preventing proper differentiation as the cells more rapidly progress through the dermis to the epidermis (15). Anti-IL-22 Ab has shown promise in the treatment of patients with atopic dermatitis (16). In contrast, IL-22 has been shown to be both protective and pathogenic in different inflammatory models of the GI tract; it is often protective in acute models and pathogenic in chronic models of inflammation (5).

IL-22 is a potent cytokine that can regulate many critical cell pathways in tissues. As such, an individual must regulate this activity not only at the molecular and protein levels of cytokine transcription and translation, but also at a biological level to control IL-22 after it has been released by immune cells into the “environment” of the individual. This is where IL-22BP enters the cytokine story.

## IL-22BP: The Fundamentals

IL-22BP was identified as a soluble receptor homolog of IL-22R soon after the identification of IL-22R as part of the receptor for IL-22 (17–19). The gene encoding IL-22BP, *IL22RA2* in humans and *Il22ra2* in mice, is encoded on chromosome 6 in humans and chromosome 10 in mice (17). The gene encoding the receptor chain IL-22R, *IL22RA1* in humans and *Il22ra1* in mice, is encoded on chromosome 1 in humans and chromosome 4 in mice (7, 8). This suggests that the gene encoding IL-22BP may have arisen from an evolutionarily distant gene duplication event. IL-22BP shares 34% sequence homology with the extracellular domain of IL-22R (20). This shared homology extends to the secondary and tertiary structures, which allows for IL-22BP to bind IL-22 in a manner that blocks IL-22-IL-22R binding.

## IL-22-IL-22BP Binding Dynamics

IL-22, encoded as a 179 amino acid protein that is an active secreted 146 amino acid protein, is a compact bundle of six anti-parallel α-helices (21). IL-22 is active as a monomer, but has been found as dimers and tetramers at high concentrations in a laboratory setting (21). Secreted IL-22BP is an approximately 24-kDa protein and forms an L-shaped structure with two fibronectin-III domains in tandem (22).

IL-22 binds to IL-22R with a high affinity and has no affinity for IL-10R2, leading to a model where IL-22 binds IL-22R, allowing binding of IL-10R2 and subsequent downstream signaling (20). IL-22 and IL-22R bind at a one-to-one ratio. IL-22BP binds to IL-22 at a site that overlaps with IL-22R, interfering with the ability of IL-22 to bind to IL-22R, and thus inhibiting cell signaling. The affinity of IL-22 and IL-22BP is K_D_ 1 pM compared with K_D_ 20 nM for IL-22 and IL-22R (20). The K_off_ rate for IL-22-IL-22BP is 4.7 days, whereas it is only a few minutes for IL-22-IL-22R (20, 22). The 10,000-fold higher affinity for IL-22 to IL-22BP than to IL-22R and the difference in K_off_ rate makes IL-22 bound to IL-22BP essentially inaccessible to IL-22R. The 2.75 Å resolution crystal structure of IL-22 bound to IL-22BP has revealed that, like IL-10 binding to IL-10R1 and IL-22 binding to IL-22R, IL-22BP contacts IL-22 using five binding loops (22).

## IL-22BP Regulation

Like IL-22, IL-22BP is primarily produced by immune cells. Unlike many immune effector molecules that are more highly expressed by activated immune cells than by their resting counterparts, IL-22BP is expressed constitutively. In healthy rodents, *Il22ra2* levels are highest in the spleen and mesenteric lymph nodes and substantial in the small intestine and colon compared with other tissues, such as the thymus, heart, bladder, and liver (23). Dendritic cell (DC) production of IL-22BP has been best characterized (23–26). A recent study using single cell RNA sequencing expanded upon past studies, showing that IL-22BP is produced by distinct subset of DCs in the GI tract (26). CD11c+ DCs within the cryptopatches and isolated lymphoid follicles respond to lymphotoxin-β with production of IL-22BP. Other identified immune cell sources include eosinophils and CD4 T cells (27, 28). IL-22BP has been shown to be produced by keratinocytes (29), which are also a source of other IL-10-related cytokines, IL-19, IL-20 and IL-24, but not IL-22 (30). Additional insight into sources of IL-22BP in humans could be gleaned from databases such as the Human Cell Atlas and Immunological Genome Project (ImmGen).

Although much of IL-22BP biology is similar between mice and humans, there is some discordance between mouse and human mRNA that encodes IL-22BP. There are three different *IL22RA2* isoforms in humans, but just one *Il22ra2* mRNA in mice, which corresponds to human isoform 2 (31, 32). Of the human isoforms, some inhibit IL-22 and some do not. IL-22BPi1 is not secreted likely due to inclusion of exon 3 and therefore fails to bind to IL-22 (31). IL-22BPi2 binds better than IL-22BPi3, suggesting that the isoforms could have distinct temporal and spatial roles in tissues (31). In contrast, the one mouse isoform produces one form of the protein, which is able to bind IL-22 (32). Furthermore, differences in binding concentrations between mouse and human IL-22-IL-22BP complexes, mouse IL-22BP binds IL-22 at high nanomolar concentrations whereas human IL-22BP-i2 can bind IL-22 at pM concentrations (20), may also impact our elucidation of IL-22-IL-22BP biology through mouse models. *In vitro* human and translational studies are undoubtedly necessary to complement mouse models of disease to better understand IL-22BP biology.

Environmental signals that control IL-22BP production include factors that induce the protein, such as retinoic acid, and factors that downregulate IL-22BP production, such as prostaglandin E2 (PGE_2_) (23, 33). In DCs, retinoic acid induces *Il22ra2*, suggesting that this active metabolite of vitamin A has further functions in addition to its well-described role in tolerizing GI tract CD103+ DCs (34, 35). PGE_2_ is a suppressor of *Il22ra2*. In skin inflammation models where *Il22ra2* is downregulated during inflammation, inhibition of PGE_2_ inhibits inflammation-mediated decreases in *Il22ra2* levels in the skin (33). Unlike IL-22, which is highly inducible in the mouse GI tract by colonization of the microbiome, colonic levels of IL-22BP are not affected by the presence or absence of commensal bacteria (36). Other environmental signals found in an *in vivo* niche are likely important, as *in vitro* differentiated DCs produce low levels of IL-22BP. The transcription factors that control *IL22RA2* and *Il22ra2* expression have not been well elucidated. There is great potential to mine databases such as the Encyclopedia of DNA Elements (ENCODE) to identify functional elements in the human genome that regulate genes involved in IL-22-IL-22BP biology.

Most studies examining *in vivo* production of IL-22BP have focused on the GI tract. Conventional DCs in the GI tract, including CD103+ DCs, which are a source of IL-22-inducing IL-23, can secrete IL-22BP (23). CD4 T cells are also a source of IL-22BP in mouse IBD models and in Crohn’s disease (CD) and ulcerative colitis (UC) patients (28). Upon inflammasome activation, *Il22ra2* levels are downregulated in the inflamed mouse colon (25). In addition to proteins involved in inflammasome activation, such as nucleotide-binding oligomerization domain, leucine rich repeat and pyrin domain (NLR) family pyrin domain containing 3 (NLRP3), apoptosis-associated speck-like protein containing a caspase recruitment domain (CARD) domain (ASC), and caspase 1, IL-18 is also an important factor in mediating IL-22BP downregulation (25). It is not clear whether inflammasome activation downregulates *Il22ra2* expression, or if it leads to death and/or trafficking of the producing cells.

IL-22 and IL-22BP frequently have inverse levels in the healthy or inflamed GI tract (25). IL-22BP levels are highest during immune homeostasis and then rapidly downregulated during inflammation. In contrast, IL-22 levels are low in healthy tissue and are then rapidly induced in ILC3s and CD4 T cells by inflammation. Although this inverse expression was observed in a DSS-mediated model of colitis and mechanical injury in mice (25), IBD patients can have elevated levels of both IL-22 and IL-22BP (28).

## IL-22BP in Health and Disease

Many tissues can be the target of IL-22, but the best studied are the skin, liver, lung, GI tract, and central nervous system (CNS). These are also the tissues for which the role of IL-22BP has been elucidated. IL-22 is a dual-natured cytokine. As such, the role of IL-22BP in inflammation can also be pro-inflammatory or protective. All known functions attributed to IL-22BP occur through its inhibition of IL-22. Many studies, many of which are cited in this review, to elucidate the role of IL-22BP have used gene-deficient mice to determine a phenotype. Under specific pathogen free conditions these mice appear healthy, but in some, but not all, inflammation models where IL-22 is important, they have observable phenotypes. When *Il22ra2^-/-^ Il22^-/-^* double-deficient mice are studied, the phenotype is ablated and the mice return to a wild-type phenotype, suggesting that the sole function of IL-22BP is to modulate IL-22 biology (25, 37).

### Skin

Keratinocytes are highly responsive to IL-22 stimulation (14). Recognition of IL-22 leads to upregulation of antimicrobial molecules, proliferation markers, and mobility proteins (10). These responses can be further modulated by the presence of other cytokines, such as IFNγ and IL-17 (38, 39). Unlike other tissues where IL-22 is often protective, IL-22 usually has inflammatory effects in the skin (40). IL-22 contributes to psoriasis pathogenesis (15, 41), but may also play a role in other inflammatory skin diseases, such as atopic dermatitis (16). In psoriasis, IL-22 promotes proliferation and mobility of keratinocytes, preventing terminal differentiation as the cells progress through the dermis to the epidermis (10, 15). This epithelial remodeling and the induction of antimicrobial peptides and chemokines contribute to inflammation.

Compared with healthy controls, psoriasis patients have upregulated levels of IL-22 and reduced levels of IL-22BP (33, 42). Furthermore, psoriatic lesions have reduced *IL22RA2* levels compared with non-lesional tissue from the same patient. This finding suggests that reduced levels of IL-22BP and higher levels of IL-22 synergize to dramatically increase the levels of bioactive IL-22 in psoriatic skin. Unlike other tissues where the immune cells are the major source of IL-22BP, in the skin IL-22BP can be produced by keratinocytes, which may allow for epithelial autoregulation in inflamed skin (29). Research with animal models of skin inflammation has revealed a protective role for IL-22BP, which is consistent with the inflammatory role of IL-22 (40). In an imiquimod-induced skin inflammation model, mice or rats deficient in IL-22BP or rats administered anti-IL-22BP Ab have exacerbated disease that is associated with increased levels of inflammatory cytokines and IL-22-induced antimicrobial peptides (29, 42). In this same skin inflammation model, treatment of mice with recombinant IL-22BP-Fc fusion protein alleviated pathology and reduced inflammatory cytokine gene expression (29). Moving forward to more translational and clinical studies, skin is an ideal tissue for initiation of trials to investigate whether recombinant IL-22BP can alleviate inflammation in chronic inflammatory disease patients, as outcomes can be easily assessed and IL-22BP has a well-defined protective role in disease.

### GI Tract

IL-22BP functions have been best studied in the GI tract. This is not unexpected, as GI tract DCs have been shown by several different groups to be the best source of the protein. In the healthy GI tract, IL-22BP prevents IL-22 from activating the cells that comprise the follicle-associated epithelium (FAE) (36). DCs within the subepithelial dome of Peyer’s patches produce IL-22BP, which counteracts IL-22 and blocks the ability of the cytokine to activate these cells. This action allows microenvironmental regulation of IL-22 activity. This activity reduces local mucin levels, antimicrobial peptide production, and fucosylation, which are usually low in the FAE to facilitate antigen sampling. Mice deficient in IL-22BP have increased levels of mucin and antimicrobial proteins, and had reduced antigen uptake of bacterial antigens into the Peyer’s patches. IL-22BP produced by GI tract DCs is also an important regulator of lipid adsorption by epithelial cells (26). Compared with wild-type mice, mice lacking IL-22BP had reduced uptake of long chain fatty acids, which led to downstream effects, including reduced serum concentration of free fatty acids, body fat, and enteric white adipose tissues (26).

Although IL-22BP-deficient mice have changes in mucin, antimicrobial peptides, and fucosylation, this does not appear to impact the microbiome. In healthy mice, the IL-22 axis regulates the composition of the microbiome (43). While IL-22-deficient mice have dysbiosis in their GI microbiomes (44), mice lacking IL-22BP do not have detectable alterations in their microbiomes (36). This finding suggests that the absence of IL-22 can have a profound effect on the mucosal immunity, but that elevated levels of bioavailable IL-22 may not have detectable effects on the microbiome.

While IL-22BP levels rapidly decrease during inflammation, the protein has well-described functions in the context of the inflamed GI tract. CD and UC patients have elevated levels of IL-22BP in their inflamed tissues compared with healthy tissues, and they have higher levels of IL-22BP than do healthy controls (23, 28). IL-22 levels are also upregulated in CD and UC patients (23, 28, 45), suggesting that even with more IL-22BP, levels of bioactive IL-22 may be elevated in these patients. Studies using mouse colitis models that recapitulate different aspects of human IBD have shown that IL-22 biology is important in colitis severity. IL-22-deficient mice have increased susceptibility to acute dextran sulfate sodium (DSS)- and T cell-mediated colitis (46). In contrast, IL-22BP-deficient mice have no detectable difference in susceptibility to acute or chronic DSS-mediated colitis (25). This finding was ascribed to the extreme downregulation of IL-22BP during DSS-mediated colitis. If a protein is usually not present during the inflammation, it would be predicted to not have a role. However, in another study, IL-22BP-deficient rats were found to be protected from acute DSS-mediated colitis (27). Like in mice, IL-22 and IL-22BP were inversely expressed in the colon; IL-22 was low and IL-22BP was high in healthy rats. Several days post-DSS, IL-22 was elevated and IL-22BP had decreased (25, 27). Rats lacking IL-22BP had increased levels of mRNA encoding for the antimicrobial peptides lipocalin 2 and β-defensin, increased numbers of mucin-producing goblet cells, and greater epithelial cell proliferation (23), suggesting more bioactive IL-22. To provide more confusion, in a T cell-mediated model of colitis, IL-22BP from T cells was found to drive disease pathology (28). These different conclusions on the role of IL-22BP in the inflamed GI tract may seem discordant, but it is likely that all results are correct. We must continue to elucidate the role of IL-22BP in the GI tract, as microenvironmental differences likely have a large impact on outcomes.

In addition to IL-22BP involvement in the inflamed GI tract, IL-22BP also has a role in the tumorigenesis of colorectal cancer (CRC). IL-22 itself has a dual role in the development of cancer, as elucidated from a mouse model of inflammatory-driven CRC. IL-22 is protective early in the model, when it helps maintain barrier integrity and lessen inflammation, thereby reducing tumors (25). Later, during wound repair of the epithelium, IL-22 helps drive more proliferation, which promotes development of tumors. In mice that lack IL-22, through the duration of the model, the protective effect dominates, and the mice have increased numbers of and larger tumors than do mice with sufficient IL-22 (25). Interestingly, the IL-22BP-deficient mice have increased tumorigenesis, suggesting that a tumorigenic role of IL-22 dominates in these mice. More recently, lymphotoxin α and lymphotoxin β were shown to induce IL-22BP in DCs, suggesting that lymphotoxin’s anti-tumor effects are mediated through IL-22BP (47). Inhibiting lymphotoxin signaling increased tumor burden in two mouse CRC models in wild-type, but not IL-22BP-deficient, mice.

### Liver

Hepatocytes are highly responsive to IL-22 (11, 48). IL-22 stimulates expression of genes encoding for anti-apoptotic proteins, such as B cell lymphoma-2 (Bcl-2), B cell lymphoma-extra large (Bcl-xL) and myeloid cell leukemia-1 (Mcl-1), and mitogenic proteins, such as cellular myelocytomatosis oncogene (c-Myc) and cyclin D1 (11). *In vivo*, IL-22 is important in liver regeneration, as mice deficient in IL-22 or downstream signaling pathways have a severely reduced ability to increase their liver mass after partial hepatectomy (49). IL-22 is also protective in many different inflammatory settings in the liver, including conA-mediated hepatitis, which is mediated by host IFNγ; liver injury induced by carbon tetrachloride or acetaminophen; ischemia-reperfusion; and viruses (50). In many of these experimental models, such as conA-mediated hepatitis, IL-22-deficient mice are extremely more susceptible than are wild-type controls, with highly elevated aspartate transaminase (AST) and alanine transaminase (ALT) levels and large regions of necrotic tissue (48). Surprisingly, IL-22BP has been shown to have a protective role in the liver during injury mediated by acetaminophen or ischemia-reperfusion (37). In both these models, *Il22ra2^-/-^* mice had increased liver injury compared with wild-type controls. In the absence of IL-22BP, excessive IL-22 induced the chemokine CXCL10 from hepatocytes, increasing the numbers of inflammatory macrophages in the liver and resulting in greater hepatocyte injury. In contrast, studies identifying polymorphisms in the *IL22RA2* gene were associated with hepatic fibrosis from schistosome or hepatitis C virus (HCV) infection (51). These genotypes were associated with higher levels of *IL22RA2* transcripts, suggesting that IL-22BP aggravates liver fibrosis, although no mechanistic experiments were performed to support this hypothesis. In the liver, IL-22BP may be produced by CD11b+ Ly6G+ cells or other myeloid-derived cells (37). Although not yet examined, resident macrophages, such as Kupffer cells, could be a local source of IL-22BP in the livers of healthy individuals. The role of IL-22BP in many liver inflammatory diseases remains to be investigated. Clearly, the liver is sensitive to IL-22. Thus, IL-22BP may be an important regulator of this cytokine and its potent effects on hepatocytes.

### Lung

IL-22 is mainly protective in models of respiratory tract inflammation (52, 53), suggesting that its binding protein has a pathogenic role in in the inflamed airways. Several recent studies have examined the functions of IL-22BP in pulmonary infection. Compared with wild-type mice, IL-22BP-deficient mice are more resistant to *Streptococcus pneumoniae* infection, but this protection is only observed at the site of initial infection in the lung and not in organs where the pathogen disseminates, such as the spleen (54). IL-22BP neutralization also protects the host against a Gram-negative pathogen, *Pseudomonas aeruginosa* airway infection (55). IL-22BP also exacerbates influenza infection (56). As observed in the inflamed GI tract, IL-22 levels increase and IL-22BP levels decrease in the lung during influenza infection, making the lung a more pro-IL-22 environment (56). Increased levels of bioactive IL-22 in IL-22BP-deficient mice lead to reduced inflammation and increased tight junctions in their lungs, which protect the mice from influenza infection (56). Further, one study examined the role of IL-22-IL-22BP in a co-infection model. IL-22BP exacerbated bacterial-viral super infection in a mouse model studying influenza infection with co-infection with either *S. pneumoniae* or *Staphylococcus aureus* (57). As for single infections, mice deficient in IL-22BP had increased lung barrier function and increased survival compared with control mice, and also had increased levels of IL-22-inducible antimicrobial peptides. Thus, the presence of IL-22BP exacerbates lung infections.

The studies examining IL-22BP in the lung have mainly focused on infection. IL-22 plays a role in non-infectious models of inflammation, such as fibrosis and asthma (58, 59). Thus, IL-22BP may also play a role in other respiratory diseases. In summary, IL-22BP regulates IL-22 bioactivity in the lung, and when IL-22BP is absent, IL-22 can provide greater protection to the host.

### Central Nervous System (CNS)

The role of IL-22 in CNS inflammation is complicated. It can be protective, pathogenic, or have no detectable function depending on the context of inflammation. The IL-22 receptor is found on some specialized cells in the CNS, including astrocytes (60), suggesting that within the CNS there are unique IL-22-targeted cells. IL-22 has been best studied in CNS inflammation related to the autoimmune disease multiple sclerosis (MS) and in animal models of this disease, such as experimental autoimmune encephalomyelitis (EAE). In MS patients, IL-22 is upregulated in their serum and peripheral blood mononuclear cells (PBMCs) compared with serum and PBMCs of healthy controls, and IL-22 levels are higher in patients with active disease than in those with inactive disease (60). EAE experiments in rodents have revealed that upon inflammation in the CNS, *Il22* is upregulated and *Il22ra2* is downregulated in the spinal cord (61). CD11b/c+ cells are the predominant source of *Il22ra2*, compared with T cells, B cells, and DCs.

In the inflamed CNS, IL-22 has been proposed to aid in blood brain barrier disruption (62), yet also have a role in mediating protection of CNS barrier integrity (63). Further complicating the role of IL-22, although IL-22 is elevated in the CNS during EAE, one study found little role for the cytokine in IL-22-deficient mice, as they had disease pathology similar to that of control mice (64). This result has also been demonstrated by other another laboratory (61). Although IL-22 has been reported to have little to no detectable role in EAE, IL-22BP does have a role that is dependent on the presence of IL-22. IL-22BP-deficient mice have reduced disease severity compared with wild-type control mice (61). IL-22BP is pathogenic due to alleviating IL-22-mediated repression of IFNγ levels (61). This phenomenon appears to be due to the effects of IL-2BP on IL-22, as mice deficient in both molecules do not have an altered phenotype compared with wild-type control mice (61). The presence of IL-22BP is proposed to mask the potential for a role of IL-22 in EAE. In addition to a functional role for IL-22BP in experimental animal models, a gene variant of *IL22RA2*, single nucleotide polymorphism (snp) rs17066096, has been independently associated with MS in several studies (65–67). Monocytes from the risk genotype rs17066096 express more *IL22RA2 in vitro* than do normal controls (61). Also, cerebrospinal fluid levels of IL-22BP correlate with MS lesions (61). Although most CNS-related studies on IL-22 and IL-22BP have focused on MS and MS-models of disease, IL-22 has been investigated in other models of CNS inflammation. IL-22 has a pathogenic role in encephalitic West Nile virus, as it promotes viral entry into the CNS *via* the blood brain barrier (63). The role of IL-22BP has not been investigated in this viral infection model. In summary, the potential role for IL-22BP in the CNS is complicated, and further studies are warranted to determine whether IL-22 biology is a viable therapeutic target for inflammation and infection in this tissue.

## Therapeutic Potential of IL-22BP

IL-22 biology is of interest to the pharmaceutical industry for development of therapeutics to combat chronic inflammatory diseases (68). Both the protective and pathogenic natures of IL-22 are being targeted. Recombinant IL-22-Fc is under trials for wound repair in ulcerative colitis flares (NCT02749630, NCT03650413, NCT03558152), acute graft-versus-host disease (NTC04539470) and COVID-19 (NCT04386616) (69). In contrast, neutralizing Abs to IL-22 are being investigated for treatment of psoriasis (70). IL-22BP has received less scrutiny as a potential drug or drug target. Studies using experimental models of inflammation provide some insight into the potential for IL-22BP as a therapeutic. As for IL-22, there may be promise in increasing IL-22BP for some diseases and blocking IL-22BP activity in other diseases. For example, in a mouse model of sepsis, injection of IL-22BP-Fc reduced disease severity (71). In contrast, pharmacological modification of IL-22BP may be an effective strategy to limit liver cirrhosis (51). Immunodepletion of IL-22BP may be possible although targeting IL-22BP to increase bioactive IL-22 levels would need to be carefully performed as depleting IL-22BP may also deplete bound IL-22. Design of a small molecule to block the interaction of IL-22BP with IL-22 may be possible as the IL-22BP-IL-22 crystal structure has been elucidated to 2.75 Å resolution with identification of critical binding residues (22).

As preventative treatments and therapeutics targeting IL-22 biology are developed, it is important to also use our knowledge to predict potential issues. Excess IL-22BP beyond physiological levels could have undesirable side effects. Reductions in bioactive IL-22 could lead to issues such as increased susceptibility to GI or pulmonary infections. However, this is somewhat unlikely, as the only described IL-22 immunodeficiency in humans is autoimmune polyendocrinopathy candidiasis ectodermal dystrophy (APECED) (72, 73). In this rare, inherited disorder, patients generate auto-antibodies to IL-22 and/or IL-17 that predispose them to chronic mucosal fungal infections, particularly *C. albicans*. Decreased levels of IL-22BP could have long-term effects on development of cancers, particularly colorectal cancer, due to long-term higher levels of bioactive IL-22 (25).

IL-22BP may have potential as a biomarker. IL-22BP levels could be used for a prognostic or predictive capacity of different inflammatory or infectious diseases. For example, patients with active IBD on anti-TNFα therapy that responded to that therapy had undetectable levels of *IL22RA2* in T cells and DCs isolated from biopsies, compared with the patients that did not respond to anti-TNFα therapy (28). IL-22BP has been discovered and validated to predict the prognosis of colorectal cancer patients; patients with high IL-22BP have greater survival than do patients with low IL-22BP (47). IL-22 biology certainly holds promise for therapeutic targeting. IL-22BP is an important target itself, or at a minimum, should be considered when targeting IL-22.

## Conclusions

IL-22BP is a fundamental example of a protein that is downregulated during inflammation, but nevertheless has a strong effect on the course of inflammation. By binding to IL-22, IL-22BP maintains immune homeostasis and limits the activity of a potent cytokine. Many tissues, especially those at the interface with the environment, respond to IL-22 stimulation to protect themselves and the host. There are still many gaps in our knowledge on IL-22BP. Understanding how IL-22BP levels are transcriptionally and post-transcriptionally regulated and the microenvironmental interactions between IL-22BP and IL-22 are two important research directions. Ongoing and future work will help elucidate these unknowns. IL-22BP allows for more exquisite regulation of a cytokine and may be key to harnessing IL-22 biology to combat chronic inflammatory and infectious diseases.

## Author Contributions

The author confirms being the sole contributor of this work and has approved it for publication.

## Funding

Research in the Zenewicz laboratory has been supported by an OUHSC College of Medicine Alumni grant, an American Heart Association Scientist Development grant (14SDG18700043), Oklahoma Center for Advancement of Science and Technology grant (HR13-003), the National Institute of General Medical Sciences of the National Institutes of Health (P20GM103447 and P20 GM134973), and funding from the Oklahoma Center for Adult Stem Cell Research (a program of the Oklahoma Tobacco Settlement Endowment Trust), Presbyterian Health Foundation, and the Stephenson Cancer Center.

## Conflict of Interest

The author declares that the research was conducted in the absence of any commercial or financial relationships that could be construed as a potential conflict of interest.

## Publisher’s Note

All claims expressed in this article are solely those of the authors and do not necessarily represent those of their affiliated organizations, or those of the publisher, the editors and the reviewers. Any product that may be evaluated in this article, or claim that may be made by its manufacturer, is not guaranteed or endorsed by the publisher.
